# Ambient pollutants, polymorphisms associated with microRNA processing and adhesion molecules: the Normative Aging Study

**DOI:** 10.1186/1476-069X-10-45

**Published:** 2011-05-21

**Authors:** Elissa H Wilker, Stacey E Alexeeff, Helen Suh, Pantel S Vokonas, Andrea Baccarelli, Joel Schwartz

**Affiliations:** 1 Cardiovascular Epidemiology Research Unit, Beth Israel Deaconess Medical Center, Boston, MA, USA; 2 Harvard School of Public Health, Boston, MA, USA; 3 VA Normative Aging Study, Veterans Affairs Boston Healthcare System and the Department of Medicine, Boston University School of Medicine, Boston, MA, USA; 4 Channing Laboratory, Brigham and Women's Hospital, Boston, MA, USA

## Abstract

**Background:**

Particulate air pollution has been associated with cardiovascular morbidity and mortality, but it remains unclear which time windows and pollutant sources are most critical. MicroRNA (miRNA) is thought to be involved in cardiovascular regulation. However, little is known about whether polymorphisms in genes that process microRNAs influence response to pollutant exposure. We hypothesized that averaging times longer than routinely measured one or two day moving averages are associated with higher soluble intercellular adhesion molecule-1 (sICAM-1) and vascular cell adhesion molecule-1 (sVCAM-1) levels, and that stationary and mobile sources contribute differently to these effects. We also investigated whether single nucleotide polymorphisms (SNPs) in miRNA-processing genes modify these associations.

**Methods:**

sICAM-1 and sVCAM-1 were measured from 1999-2008 and matched to air pollution monitoring for fine particulate matter (PM_2.5_) black carbon, and sulfates (SO_4_^2-^). We selected 17 SNPs in five miRNA-processing genes. Mixed-effects models were used to assess effects of pollutants, SNPs, and interactions under recessive inheritance models using repeated measures.

**Results:**

723 participants with 1652 observations and 1-5 visits were included in our analyses for black carbon and PM_2.5_. Sulfate data was available for 672 participants with 1390 observations. An interquartile range change in seven day moving average of PM_2.5 _(4.27 μg/m^3^) was associated with 3.1% (95%CI: 1.6, 4.6) and 2.5% (95%CI: 0.6, 4.5) higher sICAM-1 and sVCAM-1. Interquartile range changes in sulfates (1.39 μg/m^3^) were associated with 1.4% higher (95%CI: 0.04, 2.7) and 1.6% (95%CI: -0.4, 3.7) higher sICAM-1 and sVCAM-1 respectively. No significant associations were observed for black carbon. In interaction models with PM_2.5_, both sICAM-1 and sVCAM-1 levels were lower in rs1062923 homozygous carriers. These interactions remained significant after multiple comparisons adjustment.

**Conclusions:**

PM_2.5 _seven day moving averages are associated with higher sICAM-1 and sVCAM-1 levels. SO_4_^-2 ^seven day moving averages are associated with higher sICAM-1 and a suggestive association was observed with sVCAM-1 in aging men. SNPs in miRNA-processing genes may modify associations between ambient pollution and sICAM-1 and sVCAM-1, which are correlates of atherosclerosis and cardiovascular disease.

## Background

Particulate air pollution has been associated with cardiovascular morbidity and mortality, but the underlying mechanisms are not well understood. Blood markers such as intercellular adhesion molecule-1 (ICAM-1) and vascular cell adhesion molecule-1 (VCAM-1), which are expressed on cell surfaces, are markers of inflammation and endothelial function found in soluble form (sICAM-1 and sVCAM-1) within the serum [1]. Short-term exposures to particulate air pollution have been associated with higher levels of these biomarkers within populations of susceptible individuals such as asthmatic children [2], diabetics [3], and men with coronary heart disease [4], but it is not known which time windows are most pertinent and what components and sources of pollution may be most toxic. Most studies have examined acute effects in the 24 to 48 hour range, but longer averaging times of approximately one week may be more relevant to understanding the associations between particles and cardiovascular function and processes [3,5-8]. It has also been hypothesized that pollutant sources such as traffic or coal-burning power plants contribute differently in their associations with health effects and biomarker levels, but data remain limited.

Genetic and epigenetic changes have been shown to influence individual-level responses to ambient pollution for a wide variety of cardiovascular effects [9-11]. Studies have shown that polymorphisms in genes related to oxidative stress may alter susceptibility to air pollution as measured by biomarkers of endothelial and vascular function [12-16]. Interest is growing in the examination of epigenetic changes, which refer to modification of genes that are not due to changes in the DNA itself. Widely studied epigenetic marks including DNA methylation and histone modification have been associated with changes in disease status, most notably with cancer [17], but also with heart failure [18] and coronary artery disease [11,19]. Recently, epigenetic marks have also been shown to be modified in the presence of environmental factors such as traffic [20] and benzene exposure [21]. Additionally, changes in gene-specific and global DNA methylation have been observed in response to prenatal smoking exposures [22].

We recently examined associations between blood pressure and interactions between black carbon and Single Nucleotide Polymorphisms (SNPs) in microRNA (miRNA) processing genes and observed evidence of effect modification [23], but this area of research remains relatively unexplored. miRNAS are small non-coding RNAs that repress or inhibit gene expression by targeting messenger RNA (mRNA) [24,25]. It is thought that miRNAs play a role in a number of pathogenic pathways including angiogenesis [26], inflammation [27], redox signaling [28], and stress response [29]. miRNA mediation of cardiovascular disease is of interest due to recognition that dysregulation of cell-signaling pathways associated with miRNA is a key factor affecting heart disease [24,30-33]. A few studies in controlled environments have also observed cardiovascular effects due to loss of function of miRNA processing genes [34-36]. While a number of miRNAs may target numerous genes, the processing of miRNAs to maturity is regulated by a relatively small set of genes.

For this study, we investigated whether longer averaging times of particulate air pollution (PM_2.5_) seven days for fine particulate air pollution (PM_2.5_) were associated with higher levels of adhesion molecules. We also investigated whether specific sources of particles from mobile sources, represented by black carbon (BC), and sulfates (SO_4_^2^), a surrogate for fossil-fuel burning power plants, were differentially associated with these effects, and whether those effects were modified by SNPs in miRNA-processing genes.

## Methods

### Study Population

Study participants were members of the Veterans Administration Normative Aging Study. This is an ongoing longitudinal study of aging established in 1963, details of which have been published previously [37]. Briefly, the Normative Aging Study began as a closed cohort of male volunteers from the Greater Boston area aged 21 to 80 at entry, who enrolled after an initial health screening determined that they were free of known chronic medical conditions. All participants provided written informed consent and the study was approved by all participating institutions. Participants return to the study center for examination every three to five years. By 1999, when measurements of sICAM-1 and sVCAM-1 began, 668 original participants had died and a number subjects were no longer being followed, the majority because they moved out of the region following retirement. A total of 817 participants were still returning for regular examinations on a three to five year schedule. Among these participants, 811 (99.3%) were measured for sVCAM-1 and sICAM-1 in at least one visit. Of the 811 participants with cellular adhesion molecule measurements between 1999 and 2008, we restricted to the 784 of participants who reported their race as white. We chose to restrict to individuals of white race due to the very small numbers of study participants who reported being of other races (4%) and to limit the potential for population stratification.

### sICAM-1 and sVCAM-1 measurements

sICAM-1 and sVCAM-1 were measured in the serum during medical exam visits from 1999 through 2008 by ELISA assay (R & D Systems, Minneapolis, MN), with a sensitivity of 0.35 ng/mL for sICAM-1 and 2.0 ng/mL for sVCAM-1.

### SNP Selection and Genotyping

SNPs were selected based on previously published work investigating associations between genes involved in miRNA processing and disease [38,39]. These SNPs were chosen because of overlap in pathways involved in processes related to autonomic function through cell signaling, apoptosis, angiogenesis and inflammation. Although miRNAs regulate many cellular processes, often targeting multiple genes, the processing of miRNAs to maturity is thought to be controlled by a smaller subset of genes. We selected five of these genes, which have been shown to be key regulators of miRNA processing *GEMIN3, GEMIN4, DROSHA, DICER *and *DGCR8 *[40,41].

Genotyping was performed using multiplex PCR assays designed with Sequenom SpectroDESIGNER software. The extension product was then spotted onto a 384 well spectroCHIP before analysis in the MALDI-TOF mass spectrometer. Duplication was performed on 5% of the samples. We examined associations with 17 SNPs for this analysis, all of which were successfully genotyped in the majority of study participants. Three SNPs were removed because fewer than 10 participants were homozygous carriers. This left a total of 14 SNPs included in our final analyses. All SNPs were in Hardy Weinberg Equilibrium at the 0.05 level. Linkage disequilibrium (LD) was assessed using the LDPlotter tool (https://www.pharmgat.org/Tools/pbtoldplotform).

### Measurements of ambient pollution

Ambient PM_2.5 _and BC were measured hourly at a stationary monitoring site located at the top of a building one km from the examination site with a tapered element oscillating microbalance (TEOM; model 1400A, Rupprecht & Pataschnick Co, Albany NY) and aethalometer (Magee Scientific, Berkeley, CA), respectively. These monitors are operated by the Harvard School of Public Health and measure urban background pollution. Daily averages are calculated when at least ¾ the hourly values for a given day are available. We used seven day moving averages as our *a priori *time period of interest and also examined five and nine day moving averages as sensitivity analyses. The moving average is the mean exposure for the immediate time period before each examination.

SO_4_^2- ^was measured using the Harvard/EPA Annular Denuder System (HEADS) impactors for the time period 1999 - 2002. Sulfur was measured using X-ray fluourescence (XRF) (2003-2008) and converted to SO_4_^2-^. For days when both HEADS impactors and XRF were in operation, we confirmed that these two approaches produced the same results (slope = 1, R^2 ^> 0.9). For days where duplicate XRF measures were available, we used the mean of the two measures.

### Statistical methods

All statistical analyses were carried out using SAS Version 9.2 (SAS Institute, Cary, NC) and R (R Foundation for Statistical Computing, Vienna, Austria). sICAM-1 and sVCAM-1 measurements were log-transformed to improve normality in the residuals. We first investigated the main effects of exposure to PM_2.5 _on sICAM-1 and sVCAM-1 levels within the population. BC and SO_4_^2- ^were also assessed to determine whether certain sources of ambient pollution such as traffic and power plant emissions produced stronger effects. Our *a priori *hypothesis was to examine 7 day moving averages. Analyses were performed using linear mixed effects models with random intercepts to account for the correlation between repeated measures among study participants. The following potential confounders or predictors of sICAM-1 and sVCAM-1 were chosen to be included in the analysis: education (≤12 years, 13-16 years, >16 years), ≥2 servings of alcohol per day (yes/no), age at first sICAM-1 and sVCAM-1 measurement, time since first measurement, body mass index (BMI), diabetes diagnosis or fasting blood sugar >126 mg/dL (yes/no), smoking status (current, former, never), pack-years smoked, hypertension medication use (yes/no), statin use (yes/no), apparent temperature (a measure of perceived temperature) and seasonality using sine and cosine of month. To better control for infections such as influenza, we also included a term for high C-reactive protein (CRP) (>10 mg/L).

We also wanted to determine whether SNPs in miRNA processing were associated with higher levels of cellular adhesion molecules. This was done by individually adding each SNP and its SNP-by-pollutant cross-product term into the model as specified above. We then investigated whether there were main effects associated with these SNPs.

### Multiple testing and false discovery

Multiple testing is a major concern in studies of genetic epidemiology. However, many standard methods for computing adjusted p-values and false discovery rates do not incorporate the correlation that exists in repeated measures studies. Therefore, we computed adjusted p-values using a permutation test, which does not make assumptions about the underlying null distribution of test statistics, and instead uses the data and its existing correlation within subjects to estimate the appropriate null distribution. This method avoids making strong assumptions about independence of tests that would be inappropriate for our data.

We briefly describe the permutation method, which has been widely used in genetic association studies. First, we fit models of sICAM-1 and sVCAM-1, adjusting for potential confounding variables, and obtained the observed test statistic for each miRNA SNP. Next, we fit models of sICAM-1 and sVCAM-1 again, adjusting for potential confounding variables, without including the miRNA SNPs, and obtained the residuals. We then randomly permuted the SNP data and paired each miRNA profile with a random subject and their residual (thus retaining the complex correlation structure of the repeated measures study). We regressed the residuals of sICAM-1 and sVCAM-1 on this new dataset of random miRNA-subject profiles. This permutation simulation was repeated 1000 times. These permuted datasets have only random genotype-phenotype associations. Thus, the test statistics from the permuted data represent a sample from the distribution of test statistics under the null hypothesis that there is no effect for any SNP. The adjusted p-value for each miRNA SNP based on its observed test statistic was computed as

Adjusted p-values < 0.05 were considered significant.

### Sensitivity analyses

Because all participants did not have the same number of follow-up visits, and those who had the most may have been healthier than average, we assessed whether this differential selection influenced our results. We performed sensitivity analyses restricting to participants with two or more visits. In addition to testing whether five and nine day moving averages produced significantly different associations, we also examined whether adjusting for season using dichotomous variables for Winter, Spring and Summer (treating Fall as the referent category) altered results. Finally, we performed analyses excluding current smokers and individuals with high CRP levels.

## Results

Of the 784 Participants in the Normative Aging Study who provided sICAM-1 and sVCAM-1 measurements, 53 (6.8%) where missing all genotyping. Complete covariate data and all or some of the microRNA-related genotyping was available on a total of 723 Normative Aging Study participants who provided a total of 1652 study observations. The number of study visits as well as the clinical characteristics for subjects are described in Table 1. Subjects had between one and five study visits during the study period where sICAM-1 and sVCAM-1 were measured. The majority of participants provided two or three visits. Participants were elderly males, many of whom were former smokers and had been diagnosed with hypertension. Fewer visits were available for SO_4_^2- ^analyses because these measures started later and filters were missing on some days. SO_4_^2- ^measures were available on a subset of days and were matched to a total of 672 NAS participants with 1390 study visits. The clinical characteristics of participants in this subset were similar to those with PM_2.5 _and BC measures available (results not shown). Table 2 shows the associations for an interquartile range (IQR) change in the seven day moving averages of PM_2.5_, BC and SO_4_^2- ^and sICAM-1 and sVCAM-1as well as five and nine day moving averages as a sensitivity analysis. An IQR change in PM_2.5 _(4.27 μg/m^3^) was associated with 3.1% higher (95%CI: 1.6, 4.6, p < .001) and 2.5% higher (95%CI: 0.6, 4.5, p=.01) sICAM-1 and sVCAM-1 levels respectively. An IQR change in SO_4_^2- ^(1.39 μg/m^3^) was associated with 1.4% (95%CI: 0.04, 2.7) higher sICAM-1 levels, and a non-statistically significant change of 1.6% (95%CI: -0.41,3.7) higher sVCAM-1. BC at this averaging time was not significantly associated with either measure. Spearman correlations between PM_2.5 _and BC, BC and SO_4_^2-^, and PM_25 _and SO_4_^2-^were 0.62, 0.55 and 0.80 respectively.

**Table 1:** Mean±, (SD) or study n (%) of Study Participants at baseline

Population Characteristics	mean ± SD or n (%)	
Age	72.3	± 6.9

Systolic blood pressure	133.7	± 17.3

Diastolic blood pressure	78.1	± 9.3

Body Mass Index	28.2	± 4.1

sICAM-1	299.4	± 101.6

sVCAM-1	1055.2	± 360.8

Hypertension medication	411	(57)

Statin use	262	(36)

Two or more servings of alcohol per day	147	(20)

CRP > 10 ug/ml	31	(4)

Diabetes Mellitus†	139	(19)

Years of Education ≤12	210	(29)

13-16	349	(48)

>16	164	(23)

smoking status Never	204	(28)

Current	34	(5)

Former	484	(67)

packyrs	22.3	(28)

number of study visits 1	185	(26)

2	205	(28)

3	276	(38)

4	56	(9)

5	1	(<1)

† defined as diagnosis by physician or fasting blood glucose > 126 mg/dL.

**Table 2:** Percent change in sICAM-1 and sVCAM-1 scaled to an IQR change in moving average of pollutants

	sICAM-1				sVCAM-1			
**PM2.5†**	**% change**	**95% CI**		**P-value**	**% change**	**95% CI**		**P-value**

7 day	3.1	(1.6,	4.6)	<0.001	2.5	(0.6,	4.5)	0.010

5 day	3.0	(1.6,	4.4)	<0.001	2.8	(1.0,	4.7)	0.003

9 day	3.4	(2.0,	4.9)	<0.001	2.6	(0.7,	4.5)	0.007

SO4*								

7 day	1.4	(0.04,	2.7)	0.044	1.6	(-0.4,	3.7)	0.120

5 day	1.7	(0.3,	3.1)	0.017	2.2	(0.2,	4.3)	0.030

9 day	1.3	(0.1,	2.9)	0.071	1.6	(0.5,	3.7)	0.130

BC**								

7 day	-0.9	(-2.6,	1.0)	0.352	-1.5	(-3.8,	0.8)	0.204

5 day	-1.0	(-2.7,	0.7)	0.224	-0.6	(-2.8,	1.7)	0.601

9 day	-0.7	(-2.6,	1.3)	0.503	-1.2	(-3.7,	1.3)	0.338

1652 observations from 723 participants included for PM_2.5 _models. 1390 observations from 672 participants in SO_4_^2- ^models.

† 7 day IQR change in PM_2.5 _= 4.27 μg/m^3^

* 7day IQR change in SO_4_^2^- = 1.39 μg/m^3^

** 7 day IQR change in BC = 0.33 μg/m^3^

Of the 17 SNPs that were originally genotyped, three were not included in our analyses because fewer than 10 individuals were homozygous for the minor allele in our study population (rs417309 in *DGCR8*, rs197414 in *GEMIN3 *and rs3742330 in *DICER*), leaving a total of 14 SNPs for analysis. All SNPs were in Hardy-Weinberg Equilibrium at the 0.05 level. The full list of the 14 SNPs included is described in Table 3. Two SNPs had Hardy Weinberg P-values 0.1 and 0.07 are identified, but were not excluded.

**Table 3:** miRNA processing Genes and SNPs included in this study

Gene name	Rs number	Location	Alleles	Function	Amino Acid Change	HWE*≤0.1
*Gem-associated protein 3*	rs197412	chr1:112110476	T/C	Coding exon	Isoleucine/Threonine	

*(GEMIN3)[DDX20]*	rs197388	chr1:112099005	T/A	Promoter		

*DROSHA[RNASEN]*	rs10719	chr5:31437204	C/T	3' UTR		

	rs6877842	chr5:31568395	C/G	promoter		

DICER *[DICER1]*	rs13078	chr14:94626500	T/A	3' UTR		

Gem-associated protein 4	rs7813	chr17:594936	T/C	Coding exon	Cysteine/arginine	

*[GEMIN4]*	rs1062923	chr17:595817	T/C	Coding exon	Isoleucine/Threonine	

	rs3744741	chr17:595982	C/T	Coding exon	Arginine Glutamine	

	rs4968104	chr17:596255	T/A	Coding exon	Valine/Glutamic acid	

	rs910925*	chr17:596297	G/C	Coding exon	Glycine/Alanine	0.1

	rs2740348	chr17:596685	G/C	Coding exon	Glutamic acid/Glutamine	

	rs910924	chr17:602670	C/T	Promoter		

*Digeorge syndrome*	rs3757*	chr22:18479331	A/G	3' UTR		0.07

*critical region gene 8 [ DGCR8]*	rs1640299	chr22:18478359	G/T	3' UTR		

* Indicates Hardy-Weinberg Equilibrium (HWE) p-value ≤0.1.

Our primary analysis of interest was to examine gene-by-environment interactions between PM_2.5 _levels and these SNPs. Because BC was not significantly associated with sICAM-1 or sVCAM-1, and SO_4_^2- ^was missing for a large number of days in addition to being strongly correlated with PM_2.5_, we restricted our analysis of SNP associations and their interactions to models with PM_2.5_. For both sICAM-1 and sVCAM-1, particularly strong PM-by-SNP interactions were observed with the rs1062923 *GEMIN4 *SNP. These results remained significant after adjustment for multiple testing and are presented in Table 4. None of the other interactions tested approached significance (see additional files 1 and 2).

**Table 4:** Results for rs1062923-PM_2.5 _interactions 7 day moving averages†

Endothelial Marker	Number of participants	% change (95% CI)	Unadjusted p-value For interaction	Adjusted p-value
sICAM-1 homozygous carriers	31	-9.0 (-15.0, -2.5)	0.0002*	0.004*

Wild-type and heterozygotes	686	3.4 (1.9, 4. 9)		

sVCAM-1 homozygous carriers	31	-9.2 (-17.2,-0.5)	0.005	0.1

Wild-type and heterozygotes	686	2.8 (0.9, 4.8)		

†IQR change in7 day moving average of PM_2.5 _= 4.27 μg/m^3^

*models adjusted for education (≤12 years, 13-16 years, >16 years), ≥2 alcoholic drinks per day, age at first adhesion molecule measurement, time since first measurement, body mass index (BMI), diabetes diagnosi or fasting glucose >126 mg/dL, smoking status, pack-years smoked, hypertension medication use (yes/no), statin use, apparent temperature, seasonality (sine and cosine of month), CRP >10 mg/L.

We also examined the main effects of these miRNA processing SNPs. Although no SNPs met significance criteria for main effects after adjustment for multiple testing in models predicting ICAM-1, we present results for all unadjusted associations < 0.1 in Table 5. We found that one SNP in *DICER *was associated with 10% higher sICAM-1 (95%CI: -0.1, 21.1, p = 0.05). Two SNPs in *GEMIN4 *that we previously observed to be in high linkage disequilibrium (Wilker et al. 2010) were associated with approximately 6% higher sVCAM-1 levels (p = 0.03 and 0.04, unadjusted for multiple testing).

**Table 5:** Main effects of SNPs with unadjusted p-value ≤0.1

	homozygous carriers/common allele carriers	% change	95%CI		p-value
sICAM					

rs13078	21/677	10.0	(-0.1,	21.1)	0.05
					

sVCAM-1					

rs910925	116/604	6.3	(0.6,	12.3)	0.03
rs7813	116/597	6.1	(0.4,	12.1)	0.04

†unadjusted p-values are reported here. No SNPs identified in main effects analysis were statistically significant at the 0.05 level in models adjusted for multiple testing. models adjusted for education (≤12 years, 13-16 years, >16 years), ≥2 alcoholic drinks per day, age at first adhesion molecule measurement, time since first measurement, body mass index (BMI), diabetes or fasting glucose >126 mg/dL, smoking status, pack-years smoked, hypertension medication use (yes/no), statin use, apparent temperature, seasonality (sine and cosine of month), CRP >10 mg/L.

We performed a number of sensitivity analyses to test the robustness of our results. In our analyses, restricting to participants with two or more sICAM-1 and sVCAM-1 did not change our findings. Using indicator variables for season instead of sine and cosine for month of year produced similar results. Finally, we observed that neither restricting to non-smokers, nor restricting to participants with CRP < 10 mg/dL altered the results of our pollutant effects for seven day moving averages of PM_2.5_.

## Discussion

In this repeated measures study of elderly men, we observed significant associations between seven day exposures to PM_2.5 _and both sICAM-1 and sVCAM-1 levels. Additionally, SO_4_^2- ^seven day moving averages were associated with higher levels of sICAM-1 and sVCAM-1. Results from our sensitivity analyses may suggest that even longer averaging times are relevant for PM_2.5_, particularly in association with sICAM-1. We also observed evidence of effect modification by a single SNP involved in the processing of miRNA. Results were particularly strong for PM interactions with the rs1062923 SNP in *GEMIN4 *predicting both adhesion molecules examined. In both cases, carriers of the variant had lower levels of sICAM-1 and sVCAM-1.

This contrasts with another recent publication from the Normative Aging Study which examined two day moving averages of particles and only observed an association between sICAM-1 and sVCAM-1 with BC [13]. We observed significant associations for sICAM-1 in our models of seven day moving averages for both PM_2.5 _and marginally significant (p = 0.044) results for SO_4_^2-^. Previous work from our group and others has suggested that traffic pollution may adversely affect cardiovascular health and many of these studies have observed stronger effects with the components of air pollution associated with traffic particles [8,13]. The other study we are aware of which examined associations between sICAM-1 and sVCAM-1 and SO_4_^2- ^did so in a population of diabetics and did not observe a significant association [3]. However, SO_4_^2- ^measures were only available on a subset of days in that study and the n for these analyses was small (n = 61). Our results suggest that effects of PM and SO_4_^2 ^may be observed over longer time periods. Despite declines in emissions from coal-fired fire power plants, our results suggest that these emissions remain a regulatory concern which may alter endothelial function.

Data describing the effects of miRNA processing on cardiovascular processes and inflammation is relatively limited [27,42]. Even less is known about the role these processing genes play in interacting with environmental exposures. The association that we observed for the rs1062923-PM_2.5 _cross-product predicting adhesion molecule level was consistently the strongest of all interactions tested. Carriers of this polymorphism had significantly lower sICAM-1 and sVCAM-1 responses to PM_2.5 _than wild-type and heterozygous individuals. We recently observed gene-environment interactions between BC and the rs1062923 SNP in association with systolic blood pressure [23]. Previously, rs7813, also located in the *GEMIN4 *gene, which we observed to be the associated with higher sVCAM-1 levels in unadjusted models, was also associated with renal cancer in a case-control study [39]. Relatively little is known about the *GEMIN4 *gene, but it is expressed ubiquitously and is a part of a large protein complex known as the survival of motor neuron (SMN) complex that participates in the biogenesis of ribonucleoprotein complexes (RNPs) in the cytoplasm [43]. The SMN complex then enters the nucleus with the snRNPs, delivering them to serve as components of the splicing machinery for miRNA. The activity of *GEMIN4 *has also been closely associated with Galectins 1 and 3 [44]. Galectin 1 is involved in vascular smooth muscle cell proliferation and Galectin 3 has been associated with diabetic nephropathy [45] and atherogenesis [46]. Although rs1062923 does induce a non-synonomous missense mutation from isoleucine to threonine, it is not known whether it is definitively functional.

We recognize that our study is subject to a number of limitations. We have utilized stationary measures of air pollution to represent personal exposures. Prior research indicates that when examining longitudinal exposures to air pollution, most error is of the Berkson type. To the extent that it is classical, simulation studies have shown that it is highly unlikely to bias away from the null even in the presence of covariates and indicates that this exposure misclassification may lead to an underestimation of the health effects of air pollution [47]. In addition, several studies, including one conducted in the Greater Boston area, have found that longitudinal measures of ambient particulate concentrations are representative of longitudinal variation in personal exposures [48].

Misclassification of the adhesion molecules is possible, as sICAM-1 and sVCAM-1 were measured in serum, where it is derived from cleavage and shedding from endothelial cells, but factors influencing clearance of these immunologic markers remain uncertain [49]. Additionally, although ELISA assays for the analysis of sICAM-1 and sVCAM-1 are subject to variation, the day-to-day variabilities reported in our study for reference concentrations were 10% or less and these measures have previously been associated with cardiovascular disease [7,49-51]. Variation in findings for sICAM-1 and sVCAM-1 across studies may also be due to their specific expression and roles. ICAM-1 is expressed by many cell types, including endothelial cells, fibroblasts, epithelial cells, and multiple cells of hematopoietic lineage, while expression of VCAM-1 mostly occurs on atherosclerotic plaques and is limited to activation of endothelial and smooth muscle cells [52]. Varying susceptibilities of study subjects and differences in time windows studied may have also contributed to different outcomes; discrepant findings may also arise across studies because of differing sources, composition and mixtures of pollutants.

We also recognize that despite choosing covariates which we believe to be predictors of our exposure not on the causal pathway, our results may still be subject to residual confounding. However, we also included a random intercept for each subject, which should control for unmeasured, time-invariant confounders across subjects. Additionally, while this study investigates longer moving averages than have been previously associated with acute effects, it is also possible that long-term exposures may lead to chronic effects which we have not addressed here.

Finally, we also face the problem of multiple testing. On the one hand, we are testing a number of associations and acknowledge the need to be cautious about identifying false positives. However, we have selected a pathway driven approach have restricted the number of tests performed by limiting the associations under study to recessive models of inheritance. While evidence of miRNA effects in cardiovascular disease is a rapidly developing field, we have restricted our analyses to genes involved in the processing of miRNAs in order to test these novel hypotheses. Therefore, we report both our adjusted and unadjusted p-values for this analysis. Although some of the associations we report did not meet statistical significance at the more stringent cutoff value, we believe that the consistency across main effect and PM_2.5 _interaction outcomes for SNPs in *GEMIN4*, particularly rs1062923, suggests that this particular gene and its function merit further investigation.

## Conclusions

This study provides novel evidence of effect modification of the relationship between exposure to particulate matter and biomarkers of inflammation endothelial function in a population of elderly community-dwelling men. It also suggests that air pollution from SO_4_^2- ^may play a role in these processes, especially over longer averaging times. For the seven day moving averages examined, strong evidence of effect modification was found for the rs1062923 SNP in *GEMIN4*. Our results suggest that future work investigating epigenetic regulation should consider exposure to ambient pollution as a potential marker of susceptibility. By examining the association between such biomarkers and air pollution, this paper adds to the growing body of evidence that elevated levels of particulate air pollution may induce cardiovascular effects through an interrelated process of inflammation and endothelial dysfunction.

## List of abbreviations

BC: Black Carbon; BMI: Body Mass Index; CI: Confidence Interval; miRNA: microRNA; PM_2.5_: Particulate Matter <2.5 μm/fine particulate matter; sICAM-1:Intercellular Adhesion Molecule-1; SO_4_^2-^: Sulfate; sVCAM-1: Vascular Cellular Adhesion Molecule-1

## Competing interests

The authors declare that they have no competing interests.

## Authors' contributions

EW performed the statistical analyses and drafted the manuscript. SA provided statistical programming support and helped to draft the manuscript. HS participated in the design and collection of exposure measures for the study and reviewed the manuscript, PSV participated in coordinating the clinic visits of the study subjects and reviewed the manuscript. AB contributed to developing the study design and SNP selection and assisted in manuscript revision, JS conceived the study and assisted in statistical analysis as well as drafting of the manuscript. All authors have read and approved the final manuscript.

## Supplementary Material

Additional File 1 **Table S1: Effects of an IQR change in PM_2.5 _on sICAM-1 in homozygous recessive participants compared to hetero- and homozygous carriers of dominant allele**. Associations between PM_2.5 _and sICAM by SNP carrier status for all SNPs tested. Click here for file

Additional File 2 **Table S2: Effects of an IQR change in PM_2.5 _on sVCAM-1 in homozygous recessive participants compared to hetero- and homozygous carriers of dominant allele**. Associations between PM_2.5 _and sVCAM by SNP carrier status for all SNPs tested. Click here for file
