# Supplementary material for: Ambient pollutants, polymorphisms associated with microRNA processing and adhesion molecules: the Normative Aging Study
Source: Environ Health. 2011 May 21;10:45. doi: 10.1186/1476-069X-10-45 (PMC3124411; doi:10.1186/1476-069X-10-45)
Supplement: Additional File 1 — Table S1: Effects of an IQR change in PM2.5 on sICAM-1 in homozygous recessive participants compared to hetero- and homozygous carriers of dominant allele. Associations between PM2.5 and sICAM by SNP carrier status for all SNPs tested. [file 1476-069X-10-45-S1.DOC]

Table S1: Effects of an IQR change in PM_2.5_ on sICAM-1 in homozygous recessive participants compared to hetero- and homozygous carriers of dominant allele.

| SNP | Variant carrier status | gene | % change | 95%CI | | Unadjusted  p-value | Adjusted  p-value |
| --- | --- | --- | --- | --- | --- | --- | --- |
| rs1062923 | homozygous variant carriers | GEMIN4 | -9.1 | (-15.2, | -2.5) | 0.0003 | 0.004 |
|  | major allele hetero- and homozygous carriers |  | 3.4 | (1.9, | 4.9) |  |  |
| rs2740348 | homozygous variant carriers | GEMIN4 | -0.8 | (-6.2 | 5.0) | 0.18 | 0.87 |
|  | major allele hetero- and homozygous carriers |  | 3.3 | (1.7, | 4.8) |  |  |
| rs6877842 | homozygous variant carriers | DROSHA | 9.5 | (-0.4, | 20.4) | 0.26 | 0.965 |
|  | major allele hetero- and homozygous carriers |  | 2.9 | (1.4, | 4.5) |  |  |
| rs3757 | homozygous variant carriers | GEMIN4 | 5.1 | (1.3, | 9.0) | 0.27 | 0.95 |
|  | major allele hetero- and homozygous carriers |  | 2.8 | (1.2, | 4.4) |  |  |
| rs1640299 | homozygous variant carriers | DGCR8 | 1.6 | (-1.2, | 4.6) | 0.27 | 0.97 |
|  | major allele hetero- and homozygous carriers |  | 3.4 | (1.8, | 5.0) |  |  |
| rs910925 | homozygous variant carriers | GEMIN4 | 1.7 | (-1.3, | 4.9) | 0.37 | 0.994 |
|  | major allele hetero- and homozygous carriers |  | 3.4 | (1.8, | 5.0) |  |  |
| rs7813 | homozygous variant carriers | GEMIN4 | 1.7 | (-1.4, | 4.8) | 0.38 | 0.995 |
|  | major allele hetero- and homozygous carriers |  | 3.3 | (1.7, | 4.9) |  |  |
| rs3744741 | homozygous variant carriers | GEMIN4 | 7.2 | (-5.4, | 21.4) | 0.55 | 1 |
|  | major allele hetero- and homozygous carriers |  | 3.0 | (1.6, | 4.6) |  |  |
| rs197388 | homozygous variant carriers | GEMIN3 | 5.5 | (-4.3, | 16.3) | 0.64 | 1 |
|  | major allele hetero- and homozygous carriers |  | 3.0 | (1.5, | 4.5) |  |  |
| rs10719 | homozygous variant carriers | DROSHA | 2.3 | (-2.6, | 7.5) | 0.69 | 1 |
|  | major allele hetero- and homozygous carriers |  | 3.1 | (1.6, | 4.7) |  |  |
| rs13078 | homozygous variant carriers | DICER | 1.9 | (-5.1, | 9.3) | 0.77 | 1 |
|  | major allele hetero- and homozygous carriers |  | 2.9 | (1.4, | 4.5) |  |  |
| rs910924 | homozygous variant carriers | GEMIN4 | 2.48 | (-2.4, | 7.6) | 0.88 | 1 |
|  | major allele hetero- and homozygous carriers |  | 3.07 | (1. 6, | 4.6) |  |  |
| rs4968104 | homozygous variant carriers | GEMIN4 | 2.7 | (-2.4, | 8.2) | 0.95 | 1 |
|  | major allele hetero- and homozygous carriers |  | 3.1 | (1.6, | 4.6) |  |  |
| rs197412 | homozygous variant carriers | GEMIN3 | 3.2 | (-0.2, | 6. 7) | 0.97 | 1 |
|  | major allele hetero- and homozygous carriers |  | 3.0 | (1.5 | 4.6) |  |  |
