# Supplementary material for: Ambient pollutants, polymorphisms associated with microRNA processing and adhesion molecules: the Normative Aging Study
Source: Environ Health. 2011 May 21;10:45. doi: 10.1186/1476-069X-10-45 (PMC3124411; doi:10.1186/1476-069X-10-45)
Supplement: Additional File 2 — Table S2: Effects of an IQR change in PM2.5 on sVCAM-1 in homozygous recessive participants compared to hetero- and homozygous carriers of dominant allele. Associations between PM2.5 and sVCAM by SNP carrier status for all SNPs tested. [file 1476-069X-10-45-S2.DOC]

Table S2: Effects of an IQR change in PM_2.5_ on sVCAM-1 in homozygous recessive participants compared to hetero- and homozygous carriers of dominant allele.

| SNP | Variant carrier status | Gene | % change | 95%CI | | Unadjusted  p-value | Adjusted  p-value |
| --- | --- | --- | --- | --- | --- | --- | --- |
| rs1062923 | homozygous variant carriers | GEMIN4 | -9.3 | (-17.4, | -0.5) | 0.007 | 0.1 |
|  | major allele hetero- and homozygous carriers |  | 2.82 | (0.9, | 4.9) |  |  |
| rs3757 | homozygous variant carriers | GEMIN4 | 7.9 | (2.8, | 13.3) | 0.03 | 0.305 |
|  | major allele hetero- and homozygous carriers |  | 1.8 | (-0.2, | 3.8) |  |  |
| rs1640299 | homozygous variant carriers | DGCR8 | 0.4 | (-3.3, | 4.3) | 0.22 | 0.969 |
|  | major allele hetero- and homozygous carriers |  | 3.0 | (0.9 | 5.1) |  |  |
| rs197412 | homozygous variant carriers | GEMIN3 | 0.4 | (-3.9, | 5.0) | 0.28 | 0.99 |
|  | major allele hetero- and homozygous carriers |  | 2.9 | (0.8, | 5.0) |  |  |
| rs2740348 | homozygous variant carriers | GEMIN4 | -1.5 | (-8.6, | 6.1) | 0.28 | 0.99 |
|  | major allele hetero- and homozygous carriers |  | 2.7 | (0.6, | 4.7) |  |  |
| rs910924 | homozygous variant carriers | GEMIN4 | 5.0 | (-1.5, | 12.0) | 0.41 | 1 |
|  | major allele hetero- and homozygous carriers |  | 2.30 | (0.3, | 4.4) |  |  |
| rs6877842 | homozygous variant carriers | DROSHA | 8.8 | (-4.0, | 23.4) | 0.44 | 1 |
|  | major allele hetero- and homozygous carriers |  | 2.4 | (0.4, | 4.4) |  |  |
| rs10719 | homozygous variant carriers | DROSHA | 4.7 | (-1.9, | 11.7) | 0.58 | 1 |
|  | major allele hetero- and homozygous carriers |  | 2.4 | (0.4, | 4.4) |  |  |
| rs7813 | homozygous variant carriers | GEMIN4 | 1.5 | (-2.5, | 5.7) | 0.64 | 1 |
|  | major allele hetero- and homozygous carriers |  | 2.8 | (0.7, | 4.9) |  |  |
| rs910925 | homozygous variant carriers | GEMIN4 | 1.5 | (-2.5, | 5.7) | 0.66 | 1 |
|  | major allele hetero- and homozygous carriers |  | 2.7 | (0.6, | 4.8) |  |  |
| rs197388 | homozygous variant carriers | GEMIN3 | 4.5 | (-8.1, | 18.8) | 0.76 | 1 |
|  | major allele hetero- and homozygous carriers |  | 2.4 | (0.4, | 4.4) |  |  |
| rs13078 | homozygous variant carriers | DICER | 3.3 | (-5.9, | 13.4) | 0.86 | 1 |
|  | major allele hetero- and homozygous carriers |  | 2.5 | (0.5 | 4.5) |  |  |
| rs4968104 | homozygous variant carriers | GEMIN4 | 1.9 | (-4. 7, | 9.0) | 0.88 | 1 |
|  | major allele hetero- and homozygous carriers |  | 2.7 | (0. 7, | 4.7) |  |  |
| rs3744741 | homozygous variant carriers | GEMIN4 | 3.5 | (-12.4, | 22.2) | 0.92 | 1 |
|  | major allele hetero- and homozygous carriers |  | 2.5 | (0.5, | 4.5) |  |  |
